# Nucleotide Prodrug Containing a Nonproteinogenic Amino Acid To Improve Oral Delivery of a Hepatitis C Virus Treatment

**DOI:** 10.1128/AAC.00620-18

**Published:** 2018-07-27

**Authors:** Joy Y. Feng, Ting Wang, Yeojin Park, Darius Babusis, Gabriel Birkus, Yili Xu, Christian Voitenleitner, Martijn Fenaux, Huiling Yang, Stacey Eng, Neeraj Tirunagari, Thorsten Kirschberg, Aesop Cho, Adrian S. Ray

**Affiliations:** a Gilead Sciences, Foster City, California, USA

**Keywords:** GS2, GS-6620, HCV, antiviral, carboxyl esterase 1, cathepsin A, antiviral agents, carboxyl esterase, nucleotide prodrug

## Abstract

Delivery of pharmacologically active nucleoside triphosphate analogs to sites of viral infection is challenging. In prior work we identified a 2′-*C*-methyl-1′-cyano-7-deaza-adenosine *C*-nucleotide analog with desirable selectivity and potency for the treatment of hepatitis C virus (HCV) infection. However, the prodrug selected for clinical development, GS-6620, required a high dose for meaningful efficacy and had unacceptable variability due to poor oral absorption as a result of suboptimal solubility, intestinal metabolism, and efflux transport. While obtaining clinical proof of concept for the nucleotide analog, a more effective prodrug strategy would be necessary for clinical utility. Here, we report an alternative prodrug of the same nucleoside analog identified to address liabilities of GS-6620. A phosphoramidate prodrug containing the nonproteinogenic amino acid methylalanine, an isopropyl ester and phenol in the (*S*) conformation at phosphorous, GS2, was found to have improved solubility, intestinal stability, and hepatic activation. GS2 is a more selective substrate for hepatically expressed carboxyl esterase 1 (CES1) and is resistant to hydrolysis by more widely expressed hydrolases, including cathepsin A (CatA) and CES2. Unlike GS-6620, GS2 was not cleaved by intestinally expressed CES2 and, as a result, was stable in intestinal extracts. Levels of liver triphosphate following oral administration of GS2 in animals were higher than those of GS-6620, even when administered under optimal conditions for GS-6620 absorption. Combined, these properties suggest that GS2 will have better oral absorption in the clinic when administered in a solid dosage form and the potential to extend the clinical proof of concept obtained with GS-6620.

## Abstract (precis)

Delivery of pharmacologically active nucleoside triphosphate analogs to sites of viral infection is challenging. In prior work we identified a 2′-*C*-methyl-1′-cyano-7-deaza-adenosine *C*-nucleotide analog with desirable selectivity and potency for the treatment of hepatitis C virus (HCV) infection.

## INTRODUCTION

Nucleoside analogs are hydrophilic, require sequential intracellular phosphorylation to form pharmacologically active triphosphates, and often distribute suboptimally throughout the body, resulting in reduced activity and unwanted side effects. Nucleotide prodrug strategies have been heavily explored to preferentially load target tissues and bypass the often rate-limiting first phosphorylation step (1). Despite substantial effort, only a few nucleotide prodrugs have been approved for clinical use. In particular, nucleoside analogs became a focus in drug discovery for hepatitis C virus (HCV) due to their pan-genotype activity, high barrier to resistance selection, and a lack of preexisting virus with reduced susceptibility (2). However, there has been unprecedented attrition during development of nucleoside and nucleotide prodrugs for HCV due to suboptimal efficacy and off-target toxicity (3, 4). Only one nucleotide prodrug, sofosbuvir, has been approved for HCV treatment, realizing the potential of short duration therapy without the need for interferon or ribavirin across all genotypes (5).

**FIG 1:**
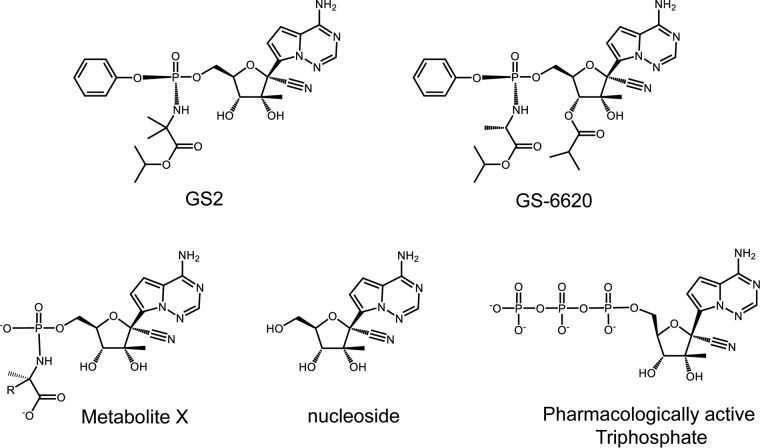
Chemical structures of GS2, GS-6620, and key metabolites. Metabolite X for GS2 R is CH_3_ and that for GS-6620 R is H.

GS-6620 is a *C*-nucleotide prodrug with potent activity against the hepatitis C virus *in vitro* (6) (Fig. 1). Its active 5′-triphosphate metabolite is a potent competitive inhibitor of HCV NS5B RNA-dependent RNA polymerase (RdRp) and has high selectivity for the viral polymerase over human DNA and RNA polymerases, including the mitochondrial RNA polymerase POLRMT (7). The double-prodrug approach employed in GS-6620 included an (*L*)-alanine-isopropyl ester and a phenol moiety that mask the two charges of the 5′-monophosphate. A 3′-isobutyryl ester was added to mask the hydrogen bond donor of the 3′-hydroxyl group and improved passive permeability and oral bioavailability (6). However, high intra- and interpatient pharmacokinetic and pharmacodynamic variability was observed in a phase I clinical study. The poor performance of GS-6620 was likely due to extensive intestinal metabolism of the prodrug by carboxyl esterase 2 (CES2), an enzyme more highly expressed in the human gastrointestinal tract than in certain species evaluated during preclinical studies, including dogs (8). As a result, the intestinal loss of the 3′ ester group likely limited the absorption potential of GS-6620. Therefore, we focused on identifying an alternative prodrug to GS-6620, with improved liver delivery following oral administration and minimal metabolism in the intestine.

It has been shown that CES1 is a key enzyme involved in hepatic activation of phosphoramidate prodrugs (8–11). Unlike CES2, which is expressed in both intestine and liver, CES1 expression is more localized to the liver in humans (12). Following ester cleavage and chemical release of the phenol moiety, another enzyme, histidine triad nucleotide binding protein 1 (HINT1), has been implicated in cleaving the P-N bond of some phosphoramidates (13). Therefore, selective CES1 cleavage followed by efficient HINT1 cleavage are desirable characteristics for promoting liver delivery.

Here, we report on an alternative prodrug to GS-6620 (GS2; Fig. 1) that was identified to improve oral delivery in the clinic. Studies were completed comparing properties predicting oral absorption (solubility, permeability, and intestinal stability) and hepatic activation of GS2 to GS-6620. Pharmacokinetic studies were completed in animals to confirm that when administered in solution or micronized suspension formulations, optimal conditions for GS-6620, that GS2 could generate at least equivalent liver levels. The combined results suggest that GS2 has the potential to be better absorbed when administered in a solid dosage form in the clinic.

## RESULTS

### Anti-HCV activity of GS2.

The 1:1 mixture of the two diastereomers at phosphorus GS2(*R*+*S*) was initially characterized for anti-HCV activity. Fractionation of GS2(*R*+*S*) using chiral column chromatography yielded slow- and fast-eluting isomers. As shown in Table 1, GS2 was approximately 4-fold more potent than the fast-eluting isomer. X-ray crystallographic determination of synthetic intermediates established the fast eluting isomer to have the (*S*) configuration at phosphorus (data not shown). Since the data for the mixture was within 2-fold of GS2's activity and showed a similar pattern of relative activity, data obtained with GS2(*R*+*S*) in some assays were reported where the single stereoisomer GS2 was not tested.

**TABLE 1:** Potency of GS2 containing diastereomeric mixture against GT1a, 1b, and 2a transiently transfected Huh-1C cells

Diastereoisomer	EC_50_^*a*^ (nM)		
	1a	1b	2a
1:1 mixture GS2(*R*+*S*)	120 ± 40	180 ± 70	71 ± 7
Slow eluting (GS2)	140 ± 60	110 ± 20	61 ± 8
Fast eluting	410 ± 110	590 ± 280	300 ± 200

a Values represent the average of ≥3 independent experiments performed in quadruplicate.

### Cell type-dependent differences in relative activity of GS2 to GS-6620.

The activities of GS-6620 and GS2 were tested in replicons for GT1a, GT1b, and GT2a generated in two different Huh-derived cell lines: stably transfected Huh-Lunet cells and transiently transfected Huh-1C cells. As shown in Table 2, GS2 was, on average, 15- and 72-fold less active in Huh-Lunet cells. Interestingly, the activity of GS2 was within an average of 3-fold of that of GS-6620 across the three genotypes in Huh-1C cells. Consistent with previously reported pan-genotype activity, similar potency for GS2 (91 to 158 nM) and relative potency to GS-6620 (within 2-fold) were obtained in chimeric replicons containing the authentic NS5b sequence in a GT1b background transiently transfected into Huh-1C cells for GT2b, GT3a, GT4a, GT5a, and GT6a (data not shown). While GS-6620 showed a maximum of 19-fold difference in activity across the 2a replicons, GS2 was found to have a nearly a 3 orders of magnitude difference in activity and was nearly inactive in Huh-Lunet cells (EC_50_, 25,000 nM). Both GS-6620 and GS2 form the same pharmacologically active triphosphate, and these results led us to suspect that activation by distinct hydrolases differentially expressed in the various cell lines strongly modulates the *in vitro* antiviral potency.

**TABLE 2:** Cell-dependent activity of GS2 against HCV replicons

HCV replicon system^*a*^ and genotype	EC_50_^*a*^ (nM)		Fold (GS2/GS-6620)
	GS-6620	GS2	
Stably transfected Huh-Lunet cells^*b*^			
1a	110 ± 50	380 ± 150	3.5
1b	300 ± 140	2,200 ± 1,300	7.2
2a	500 ± 160	25,000 ± 11,000	50
Transiently transfected Huh-1C cells			
1a	41 ± 19	140 ± 60	3.5
1b	35 ± 15	110 ± 20	3.1
2a	27 ± 10	61 ± 8	2.3

a All cells were treated for 3 days. EC_50_s represent averages ± standard deviations from >3 independent experiments.

b The stable replicon cell lines were individual clones selected from transfected Huh-Lunet cells.

### Western analysis for expression of CatA and CES1 in different cells.

**FIG 2:**
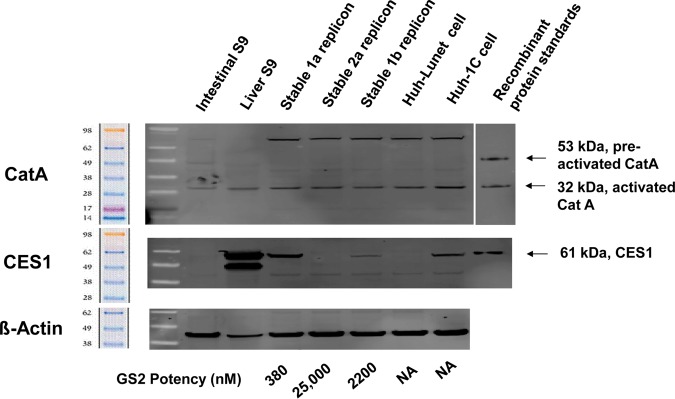
Comparison of the HCV replicon activity of GS2 to expression of the hydrolases CatA and CES1. Protein levels were visualized by Western blotting. The EC_50_s for GS2 are 380, 25,000, and 2,200 nM for 1a, 2a, and 1b replicons, respectively. The stable replicon cell lines were individual clones selected from transfected Huh-Lunet cells. Data were from *n* = 3 to 5 independent experiments done in triplicate. NA, not applicable.

The relative expression levels of hydrolases CatA and CES1 were analyzed in human intestinal S9 fraction, human liver S9 fraction, and different Huh-derived replicon cells, including stably transfected 1a, 2a, and 1b Huh-Lunet cells and untransfected Huh-Lunet and Huh-1C cells (Fig. 2). CatA expression levels in different cells were similar; however, the levels of CES1 varied greatly among different cell types. The higher level of CES1 in 1a replicon stably transfected Huh-Lunet cells and transiently transfected Huh-1C cells correlated with increased GS2 potency in these cells (Table 2). As expected, the liver S9 fraction showed high levels of CES1 expression, whereas it was undetected in the intestinal S9 fraction (12). Taken together, these results suggest that Huh-1C cells have a hydrolase expression profile more similar to that of human liver, including the expression of high levels of CES1, and that the hydrolase levels in stably transfected Huh-Lunet cells differ across the different replicons.

### Intracellular activation of GS-6620 and GS2 in cells with differential expression of CES1.

**FIG 3:**
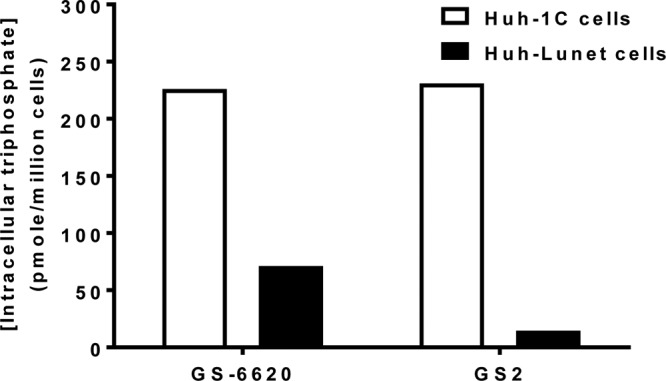
Triphosphate level of prodrugs GS-6620 and GS2 in CES1-high (Huh-1C; open bar) and -low (Huh-Lunet; filled bar) cells. Cells were incubated with 10 μM the nucleotide prodrugs for 1 h. This study was done with GS2(*R*+*S*), which showed cell loading within 2-fold of the pure isomer GS2 under these assay conditions. Representative data are shown here with averages from duplicate measurements. There is no difference between GS-6620 and GS2 in 1C cells (*P* = 0.82) and significantly lower levels of triphosphate for GS2 than GS-6620 in Lunet cells (*P* = 0.05).

In order to correlate triphosphate levels with CES1 expression levels, formation of the pharmacologically active triphosphate metabolite was determined in cells with low (Huh-Lunet) and high (Huh-1C) CES1 expression. These levels were measured following 24 h of incubation with 10 μM either GS-6620 or GS2(*R*+*S*). As shown in Fig. 3, metabolism of the two prodrugs was similar in Huh-1C cells, but triphosphate levels from GS2(*R*+*S*) were 16-fold lower in Huh-Lunet cells. These results correlate with the relative potency observed in Huh-1C and Huh-Lunet cells and further suggest the primary role of CES1 in the activation of GS2.

### ADME profiling of GS2.

The *in vitro* absorption, distribution, metabolism, and excretion (ADME) profile of GS2 relative to GS-6620 is summarized in Table 3. GS2 has substantially higher solubility than GS-6620. In particular, solid GS2 showed >30-fold higher thermodynamic solubility in fed-state simulated intestinal fluid than GS-6620. This improvement in thermodynamic solubility was observed across solid forms and in different buffers (data not shown). The difference in solubility for GS2 was also less than 30% between simulated intestinal fluid mimicking fed and fasted conditions. This improved solubility resulted in higher A-B permeability through Caco-2 monolayers for GS2 than GS-6620 when incubated at their respective thermodynamic solubility limits. When permeability was tested at similar concentrations, GS-6620 had higher permeability than GS2. However, at lower concentrations the observation of reduced forward permeability and increased efflux ratio illustrates that GS2 is subject to saturable efflux transport that is partially overcome by its high solubility.

**TABLE 3:** Improved *in vitro* ADME parameters of GS2 compared to GS-6620

Property	Condition	Value for:	
		GS-6620	GS2
Solubility (concn; μM)			
Kinetic	PBS (pH 7.0)	46	>100
	HCl (pH 1.0)	87	>100
Thermodynamic (simulated intestinal fluid; fed)		7.8	430
Absorption (cm/s, ×10^6^)	Papp A→B	1.8	2.6
Caco-2 permeability^*a*^	Papp B→A	11	6.7
Efflux ratio	B→A/A→B	5.8	2.5
Metabolic stability in intestinal subcellular (S9) fraction (half-life; min)			
Hamster		<2	67
Rat		8.8	170
Dog		33	550
Monkey (cynomolgus)		5.6	230
Human		17	570
Metabolic stability in hepatic subcellular (S9) fraction (predicted hepatic extraction; %)			
Hamster		97	93
Rat		97	86
Dog		73	42
Monkey (cynomolgus)		95	91
Human		93	87
Plasma stability (half-life; min)			
Hamster		120	Stable^*b*^
Rat		<2.0	5.0
Dog		430	Stable
Monkey (cynomolgus)		80	Stable
Human		370	Stable

a Permeability measured at thermodynamic solubility limit in fed simulated intestinal fluid. GS2 permeability when tested at 10 μM is 0.40 × 10^−6^ and 9.3 × 10^−6^ cm/s in the A→B and B→A directions, respectively.

b Stable reflects a half-life of >600 min.

GS2 showed higher stability in intestinal S9 from hamster, rat, dog, monkey, and human. While GS-6620 had a half-life of <30 min in all species except dog, GS2 had a half-life of greater than 1 h in all species and was numerically most stable in human intestinal S9 (Table 3). The stability of GS2 was 35-fold greater than that of GS-6620 in human intestinal S9. In contrast, GS2, like GS-6620, was highly unstable in hepatic S9 fractions across species. Similar to GS-6620, GS2 was stable (half-life of >1 h) in plasma from hamster, dog, monkey, and human but unstable in rat plasma due to higher levels of circulating esterase activity (14). The results from plasma and extract stability studies suggest that GS2 will suffer less intestinal metabolism than GS-6620 and will be more selectively activated in the liver.

### Intracellular activation of GS2 in human primary hepatocytes.

**FIG 4:**
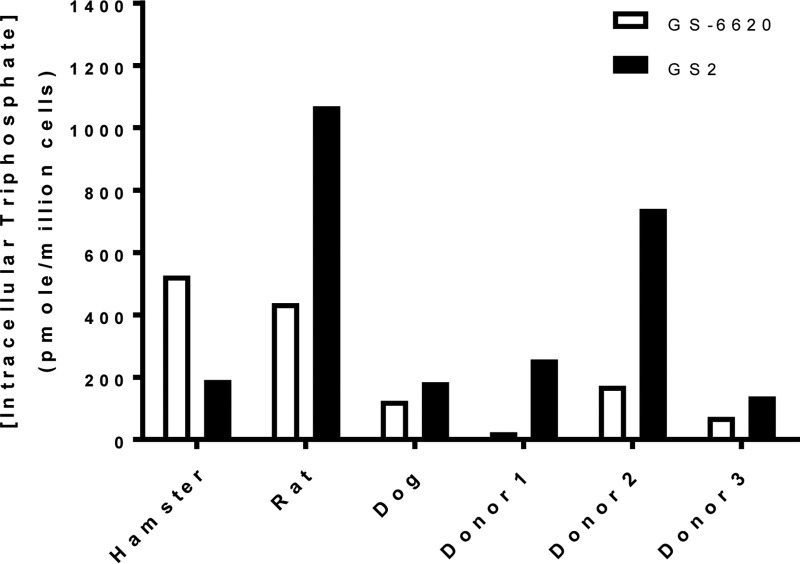
Maximal intracellular triphosphate concentrations following 1-h pulse incubations with primary hepatocytes from hamsters, rats, dogs, or humans (isolated from 3 separate human donors) with 10 μM GS-6620 (open bars) or GS2 (filled bars). Values are the means from duplicate samples collected for each individual primary hepatocyte donor.

GS2 was efficiently activated in freshly isolated primary hepatocytes *in vitro*. Following a 1-h incubation with primary human hepatocytes, GS2 showed 1.9- to 11-fold higher levels of triphosphate metabolite than GS-6620 in cells from 3 separate donors (Fig. 4). Of the animal hepatocytes tested, only rat showed relative activation of the two prodrugs similar to that of human, with dog showing similar levels of triphosphate and hamster metabolism favoring GS-6620. It is important to take into account the species difference in hepatocyte activation when interpreting liver levels in the animal pharmacokinetic studies presented below.

### Metabolism by hydrolases.

Human CatA and CES1 were studied for their specific activity for cleavage of the isopropyl ester in GS-6620 and GS2 and the 3′-ester in GS-6620. As shown in Table 4, CatA showed 11-fold lower activity toward GS2 than the GS-6620-containing diastereomeric mixture, while CES1 hydrolyzed GS2 48-fold more efficiently than GS-6620. In contrast, CES2 was unable to cleave the isopropyl ester of either prodrug. The 3′-ester cleavage of GS-6620 could be catalyzed by both CES1 and CES2. Unlike GS-6620, GS2 lacks a 3′-ester and avoids cleavage by intestinally expressed CES2.

**TABLE 4:** Specific activity for ester cleavage of GS-6620 and GS2 by CatA, CES1, and CES2

Compound^*a*^	Amidate ester cleavage (pmol/min · μg)			3′-Ester cleavage (pmol/min · μg)	
	CatA	CES1	CES2	CES1	CES2
GS2	385	91.0	Stable	NA^*b*^	NA
GS-6620	4,163	1.9	Stable	1.6	60.1

a Specific activity cannot be compared between enzymes because the assays have been optimized differently. GS-6620-containing diastereomeric mixture was used.

b NA, not applicable.

HINT1 was studied for its specific activity for catalyzing cleavage of the P-N bond in the respective intermediate metabolite X of GS-6620 and GS2. Cleavage of the P-N bond is often a limiting factor based on the observation that metabolite X was a major circulating species after administration of GS-6620 (8). HINT1 showed 5.7-fold higher specific activity toward metabolite X of GS2 (341 pmol/min μg) than that of GS-6620 (60 pmol/min μg). The enhanced cleavage by HINT1 in part can explain the more efficient liver activation.

### Absorption and hepatic extraction in portal vein-cannulated dogs.

**FIG 5:**
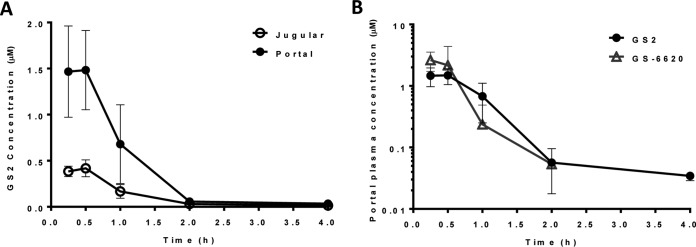
Portal and jugular plasma concentrations versus time for GS2 following oral administration to portal vein-cannulated male beagle dogs (5 mg/kg; solution) (means ± standard deviations, *n* = 3). (A) The portal (closed circle) and jugular (open circle) plasma concentrations are shown. GS2 showed an *F_a_* (faction absorbed) value of 52% and an *E_H_* (hepatic extraction) value of 75%. (B) Portal plasma concentration versus time for GS2 (5 mg/kg; solution) and GS-6620 (5 mg/kg; suspension of micronized material) following oral administration to portal vein-cannulated male beagle dogs (means ± standard deviations, *n* = 3).

The absorption and subsequent hepatic extraction of GS2 were studied in pentagastrin-pretreated and portal vein-cannulated dogs. Following oral administration of GS2, 51.6% of the dose was absorbed as intact prodrug, generating high portal vein concentrations. Extensive extraction by the liver resulted in minimal systemic exposure (Fig. 5A).

The portal vein profile and pharmacokinetic parameters following oral administration to pentagastrin-pretreated and portal vein-cannulated dogs of GS2 were compared to those of GS-6620 (Fig. 5B and Table 5). Plasma exposure to GS-6620 was transient relative to that of GS2, yielding higher portal maximum concentration of drug in serum (*C*_max_) yet decreasing to an undetectable concentration by 2 h postdose. While the maximal portal concentration obtained by GS2 was approximately 2-fold lower than that of GS-6620, all other pharmacokinetic parameters were within 2-fold due to its relatively prolonged exposure.

**TABLE 5:** Mean pharmacokinetic parameters for GS2 and GS-6620 following oral administration to pentagastrin-pretreated portal vein-cannulated male beagle dogs^*d*^

Prodrug	*C*_max_ portal (μM)	AUC_0–_*_t_* portal (μM · h)	*C*_max_ jugular (μM)	AUC_0–_*_t_* jugular (μM · h)	*F_a_*^*a*^ (%)	*E_H_*^*b*^ (%)	*F*^*c*^ (%)
GS2	1.61 ± 0.35	1.77 ± 0.26	0.43 ± 0.09	0.44 ± 0.09	52	75	13
GS-6620	3.65 ± 0.86	1.68 ± 0.80	0.62 ± 0.43	0.33 ± 0.28	79	80	15

a Fraction absorbed (*F_a_*) was calculated by comparing the dose-normalized portal vein exposure (AUC_0–_*_t_*) following oral administration to the AUC_0–_*_t_* observed following a 0.5-mg/kg intravenous infusion (30 min) of 0.34 ± 0.04 μM · h and 0.20 ± 0.04 μM · h for GS2 and GS-6620, respectively. Exposure values for intravenous infusion are the means ± SD (*n* = 3).

b Hepatic extraction (*E_H_*) was calculated using the equation 100% × (AUC_0–_*_t_* portal − AUC_0–_*_t_* jugular)/AUC_0–_*_t_* jugular.

c Oral bioavailability (F) was calculated by comparing the dose-normalized jugular vein AUC_0–_*_t_* following oral administration to AUC_0–_*_t_* observed following intravenous infusion.

d GS2 was used at 5 mg/kg in solution, and GS-6620 was used at 5 mg/kg in suspension of micronized material.

### Liver pharmacokinetics in hamster, rat, and dog.

**FIG 6:**
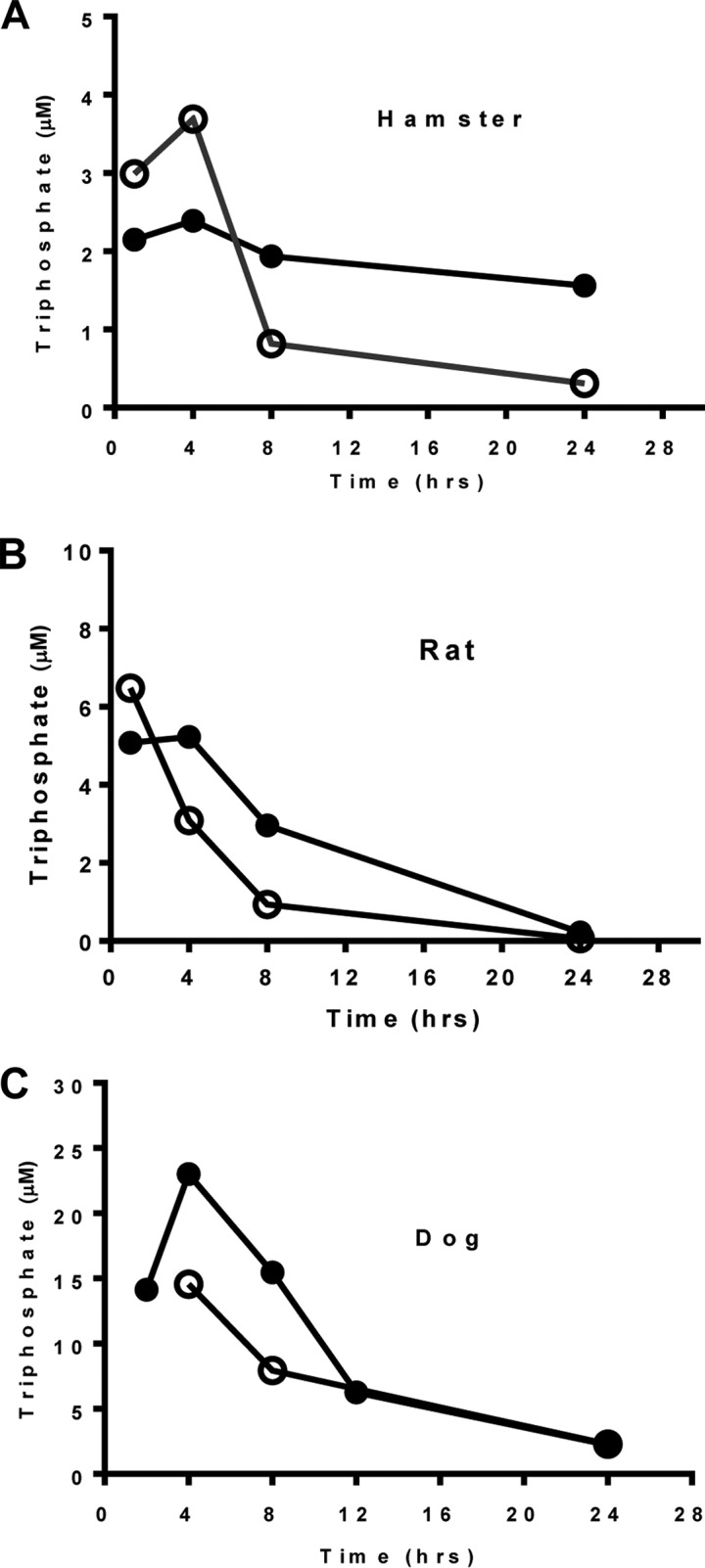
Liver levels of the active triphosphate in multiple animal species. (A) Per os dosing in Golden Syrian hamster of either GS-6620-containing mixture (10.6 mg/kg; open circle) or GS2(*R*+*S*) (9.64 mg/kg; closed circle); these doses contained 5-mg/kg equivalent of parent nucleoside. (B) Per os dosing in Sprague-Dawley rat of either GS-6620-containing mixture (21.11 mg/kg; open circle) or GS2(*R*+*S*) (50 mg/kg, dose normalized to 10 mg/kg parent nucleoside; closed circle). (C) Per os dosing in beagle dog of either GS-6620 (7.7 mg/kg, dose normalized to 2.59 mg/kg parent nucleoside; open circle) or GS2 (5 mg/kg, equivalent to 2.59 mg/kg parent nucleoside; closed circle). The values represent averages from terminal liver collections from two animals per time point.

Liver levels of the common pharmacologically active triphosphate were measured at several time points through 24 h, following oral administration of the GS2(*R*+*S*) or diastereomeric mixtures containing GS-6620 in hamsters and rat, alongside data from the oral administration of GS2 and GS-6620 in dogs (Table 6 and Fig. 6). In rodents, GS2 resulted in higher and more persistent levels of triphosphate than GS-6620. For example, in hamsters, GS-6620 achieved a 1.5-fold higher *C*_max_ at 4 h than GS2, but GS2 had a trough concentration at 24 h that was 5-fold higher than that of GS-6620. Similar to results reported with sofosbuvir (15), triphosphate formation in dogs was significantly greater than that in rodents. As shown in Fig. 6C, dose-normalized liver levels of the triphosphate were 60% higher between 4 and 8 h following oral administration of GS2 relative to GS-6620 in dogs. Overall, the dose-normalized area under the concentration-time curve from 0 to 24 h (AUC_0–24_) for liver triphosphate for GS2 was 1.5-, 1.8-, and 1.4-fold higher than that of GS-6620 in hamster, rat, and dog, respectively (Table 6).

**TABLE 6:** Liver pharmacokinetic parameters for the active triphosphate in multiple animal species following oral administration of GS2 or GS-6620

Species and prodrug	AUC_0–24_ (μM · h)	*C*_max_ (μM)	*C*_trough_ (μM)
Hamster^*a*^			
GS2	44.5	2.39	1.56
GS-6620	29.6	3.69	0.31
Rat^*b*^			
GS2	59.9	5.23	0.22
GS-6620	33.6	6.48	0.06
Dog^*c*^			
GS2	223	23.0	2.24
GS-6620	156	14.6	2.30

a Golden Syrian hamsters were administered a mixture containing either GS-6620 (10.6 mg/kg) or GS2(*R*+*S*) (9.64 mg/kg). These doses correspond to a 5-mg/kg equivalent of parent nucleoside.

b Sprague Dawley rats were administered either GS-6620-containing mixture (21.11 mg/kg; 10-mg/kg equivalent of the parent nucleoside) or GS2(*R*+*S*) (50 mg/kg; dose-normalized to 10 mg/kg parent nucleoside).

c Beagle dogs were orally administered either GS-6620 (7.7 mg/kg, dose normalized to 2.59 mg/kg parent nucleoside) or GS2 (5 mg/kg, equivalent to 2.59 mg/kg parent nucleoside). Values represent averages from terminal liver collections from two animals per time point.

## DISCUSSION

While a number of nucleotide prodrug kinase bypass strategies have been successful in improving *in vitro* activity, identification of prodrugs with acceptable pharmacokinetics, including intestinal absorption and tissue delivery, has been decidedly more challenging (1). In particular, nucleotide prodrugs have to survive the intestinal lumen, where enzymes with carboxylesterase activity, including chymotrypsin, exist and then pass through a layer of enterocytes expressing CES2, among other hydrolases, to reach the liver (16). Compounding these issues, nucleotide prodrugs are often substrates for intestinal efflux transporters, like P-glycoprotein, resulting in increased residence time to allow for hydrolase degradation (17). While selected for its improved intrinsic permeability, GS-6620 suffered from suboptimal solubility, intestinal efflux, and CES2-mediated instability that, when combined, may explain its poor performance in the clinic (8).

We describe here an alternative prodrug to GS-6620 identified to more efficiently deliver the same nucleoside analog triphosphate into the liver following oral administration. The nonclinical profile of GS2 suggests its potential to address the observed limitation of GS-6620 that required higher-dose administrations to drive antiviral activity. Nucleotide prodrugs show concentration-dependent absorption, requiring high concentrations in order to saturate two major barriers to their intestinal absorption: esterase cleavage and efflux transport. The thermodynamic solubility of GS2 was substantially improved relative to that of GS-6620 across a range of conditions. Enhanced solubility is predicted to provide increased absorption with reduced variability for GS2 in human subjects. In fact, GS2 had higher A-to-B permeability and reduced efflux than GS-6620 when incubated at the prodrugs' respective aqueous solubility limits. GS2 is also 35-fold more metabolically stable than GS-6620 in the human intestinal S9 fraction, suggesting reduced loss of parent to metabolism during absorption.

We knew that identifying an alternative prodrug to GS-6620 would be challenging. GS-6620 was originally selected from a large number of prodrugs primarily due to its improved permeability in Caco-2 cells and high absorption in dog (6). In particular, the 3′-ester markedly enhanced passive permeability. For example, the corresponding l-alanine prodrug lacking a 3′-ester had markedly lower Caco-2 permeability and an *F_a_* of only 10% in dogs (6). However, the 3′-ester also reduced solubility and introduced a metabolic soft spot for intestinal esterase cleavage. As we have previously reported, lower levels of GS-6620 and its metabolites were observed in the clinic than would have been predicted based on dog studies, likely due to higher intestinal hydrolase activity and CES2 expression in human (8). The addition of a single methyl group in the nonproteinogenic amino acid resulted in GS2 having an *F_a_* in dog more than 5-fold higher (52%; Table 5) than that of its corresponding l-alanine-containing prodrug while maintaining high solubility and improved intestinal stability. While GS2 was unable to match the intestinal absorption of GS-6620 in dog, these studies show GS2 to be well absorbed, and based on its solubility and intestinal stability, the high absorption in dog observed under optimal conditions (solution formulation and acidic stomach pH) are more likely to be maintained in the clinic by GS2 (6).

In addition to selecting a prodrug with intestinal absorption as high as possible in the absence of a 3′-substitution, efforts were also made to select a prodrug that is more efficiently metabolized in human hepatocytes once absorbed. This was of particular importance because the goal was to substantially lower the dose from that required to observe targeted activity for GS-6620 in the clinic (450 to 900 mg twice daily depending on the formulation). While GS2 showed a remarkable level of cell background-dependent variability in replicon activity, it was found to be, on average, 5-fold more efficient at loading primary human hepatocytes *in vitro*. Mechanistic studies with enzyme preparations established that improved activation in hepatocytes was a product of both more efficient initial isopropyl ester cleavage by CES1 and phosphoramidate cleavage of metabolite X by HINT1. As freshly isolated primary hepatocytes are the most relevant cell type that maintains CES1 activity in the intact liver, these results suggest that absorbed GS2 reaching the liver will be more efficiently activated. Further, the more selective cleavage by CES1 should result in limited exposure to other tissues and decreased potential side effects.

Oral administration of GS2 resulted in at least a 1.4-fold higher dose-normalized liver exposure of active triphosphate in hamsters, rats, and dogs than GS-6620. A similar result was also observed in cynomolgus monkeys (data not shown). Notably, the advantage in liver levels for GS2 was observed in two species: (i) in hamsters, where GS-6620 was more efficiently metabolized in isolated hepatocytes *in vitro*, and (ii) in dogs, where GS2 was not able to match the intestinal absorption of GS-6620 and had similar activation in isolated hepatocytes *in vitro*. In particular, liver levels of pharmacologically active triphosphate of GS2 compared favorably to those reported for sofosbuvir in rats and were within 2-fold in dogs (15).

In conclusion, GS2 was found to have markedly improved solubility, intestinal stability, and hepatic activation relative to those of GS-6620. While still less intrinsically permeable than GS-6620, as reflected in a lower fraction absorbed in dogs, these properties should result in better performance in humans administered a solid dosage form due to the improved solubility and stability for CES2, a more highly expressed isoform in human than dog. Improved hepatic activation should serve to further reduce the required dose needed to obtain targeted antiviral activity. Taken together, these properties should allow for GS2 to achieve sufficient and consistent liver loading of the pharmacologically active triphosphate to drive the antiviral effects that were only inconsistently observed with GS-6620 when given at high doses.

## MATERIALS AND METHODS

### Materials.

GS-6620, GS2, metabolite X, nucleoside, and its 5′-triphosphate active metabolite were synthesized at Gilead Sciences, Inc. (Foster City, CA). The hydrolase cathepsin A (CatA) was purified from peripheral blood mononuclear cells (PBMCs) according to a previously published procedure (18). CES1 and CES2 were purchased from R&D Systems (Minneapolis, MN), and HINT1 was provided by W. Wagner (University of Minnesota, Minneapolis, MN). Hamster, rat, dog, monkey, and human plasma samples were obtained from BioreclamationIVT (Baltimore, MD). Intestinal and hepatic S9 fractions from hamsters, rats, dogs, monkeys, and humans were obtained from Celsis *In Vitro* Technologies (Baltimore, MD).

### Cells.

Primary hepatocytes from humans, hamsters, dogs, and monkeys were purchased from BioreclamationIVT in 12-well plates seeded at confluence (0.88 × 10^6^ cells/well). Huh-Lunet cells were obtained from ReBlikon GmbH (Mainz, Germany) (19). Huh-1C was derived from a GT1a replicon clone in which HCV replication was cured. All Huh-Lunet- and Huh-1C-based replicon cell lines were cultured in Dulbecco's modified Eagles' medium (DMEM) with GlutaMAX (Invitrogen, Carlsbad, CA) supplemented with 10% fetal bovine serum (FBS; HyClone, Logan, UT) and 100 U/ml penicillin (Invitrogen). Replicon cell lines containing genotype 1a (H77) and 2a (JFH1) were created as previously reported (20–22). Caco-2 cells were maintained in DMEM with sodium pyruvate, GlutaMAX supplemented with 1% penicillin-streptomycin, 1% nonessential amino acids, and 10% FBS in an incubator set at 37°C, 90% humidity, and 5% CO_2_.

### GS2 and its diastereomeric mixture, GS2(*R*+*S*).

GS2 is a single diastereomeric prodrug, as shown in Fig. 1. Initially, the 1:1 mixture of the two diastereomers, including GS2, was used to characterize the prodrug and is denoted GS2(*R*+*S*).

### HCV replicons.

GT1a, GT1b, and GT2a are authentic subgenomic replicons of the indicated strain. Creation of these HCV replicons was reported previously (7).

### Transient transfection.

RNA was transcribed *in vitro* using a MEGAscript T4 kit (Ambion, Austin, TX) and transfected into Huh-Lunet and −1C cells using published methods (21, 22).

### Enzymatic assays.

Biochemical assays studying prodrug cleavage have been described previously (8). In brief, the cleavages of GS2 by CATA, CES1, CES2, and HINT1 were assessed *in vitro*. The reaction substrates and products were monitored over time by high-performance liquid chromatography (HPLC).

### Stability in blood plasma and S9 fractions.

Blood plasma and S9 stability was studied as described previously (8). In brief, the compounds were incubated at 2 μM in hamster, rat, dog, monkey, or human plasma samples for up to 4 h at 37°C. For S9 stability, the compounds were incubated at 2 μM in hamster, rat, dog, monkey, or human intestinal and hepatic S9 fractions for up to 90 min at 37°C in the presence of NADPH and uridine 5′-diphosphoglucuronic acid trisodium salt (UDPGA) (Sigma-Adrich). The samples were analyzed by liquid chromatography coupled to triple-quadrupole mass spectrometry (LC-MS/MS).

The data (analyte-to-internal standard peak area ratio) were plotted on a semilog scale and fitted using an exponential fit. The half-life (*t*_1/2_) was determined assuming first-order kinetics.

### Intracellular activation of GS-6620 and GS2 in Huh-Lunet, Huh-1C, and primary hepatocytes.

Methods for determining intracellular activation in cell lines and primary hepatocytes has been described in detail (8). In brief, Huh-Lunet and Huh-1C cells were seeded in 12-well plates at confluence (0.44 × 10^6^ cells/well), and primary human hepatocytes from three different donors and animal hepatocytes from hamster, rat, and dog were seeded in 12-well plates at confluence (0.88 × 10^6^ cells/well). Ten μM GS2 or GS-6620 was incubated for 24 h in Huh-Lunet and Huh-1C cells and for 1 h in primary hepatocytes. The intracellular metabolites were extracted and analyzed by LC-MS/MS.

### Bidirectional permeability assay.

Caco-2 cells between passage 43 and 71 were grown to confluence for ≥21 days on 12- or 24-well polyethylene terephthalate plates (BD Biosciences). The bidirectional permeability of compounds was tested as described previously (8). In brief, the donor well contained Hanks' balanced salt solution (HBSS), 10 mM HEPES (pH 6.5), and 15 mM glucose. The receiver well contained HBSS (pH 7.4) and 1% bovine serum albumin (BSA). The cells were dosed on the apical (A) or basolateral (B) side to determine forward (A to B) and reverse (B to A) permeability. Media from the donor and receiver wells were analyzed by LC-MS/MS.

### Solubility assays.

As described previously (8), kinetic solubility was measured by diluting a 10 mM dimethyl sulfoxide (DMSO) stock solution of the test compound down to 100 μM in PBS (pH 7.0) or 0.1 N HCl (pH 1.0). The samples were incubated at 37°C for 1 h, followed by centrifugation at 2,800 relative centrifugal force (RCF) for 30 min at room temperature. Thermodynamic solubility was determined with the crystalline-free base of GS-6620 or amorphous GS2 using the shaking flask method. The concentrations of the compounds in supernatants were determined by HPLC.

### Animal pharmacokinetics.

Plasma and liver pharmacokinetic studies were completed as described previously (8). Briefly, intact Golden Syrian hamsters, Sprague-Dawley rats, and beagle dogs (intact and portal vein cannulated) were orally administered a single dose of GS2 or GS2(*R+S*) in solution formulations at between 5 and 50 mg/kg of body weight. Similarly, studies were completed by administering GS-6620 or its 1:1 diastereomeric mixture in solution or micronized suspension formulations at between 5 and 21.1 mg/kg. Animal doses were chosen to correspond to surface area-adjusted human doses targeted for clinical development (150 to 500 mg). In order to understand hepatic extraction (*E_H_*), fraction absorbed (*F_a_*), and oral bioavailability (*F*), intravenous (i.v.) and oral (p.o.) studies were conducted with GS2 and GS-6620 in intact and portal vein-cannulated dogs. Intravenous dosing used solution formulations at 0.5 mg/kg of the respective prodrugs. Animals were housed and handled in accordance with the *Guide for the Care and Use of Laboratory Animals* (23). The protocols were reviewed and approved by the Institutional Animal Care and Use Committees (IACUC).

### Bioanalytical methods for plasma and liver samples.

Plasma samples were prepared through protein precipitation extraction by adding acetonitrile to a final concentration of 70% containing internal standard (5-iodotubericidin). Samples were dried completely under a stream of nitrogen at 40°C and reconstituted with 0.2% formic acid in water to 3 times the original volume of plasma for LC-MS/MS analysis. Liver samples were prepared by sectioning into smaller pieces and collecting into preweighed 15-ml conical tubes kept on dry ice. Ice-cold extraction buffer (0.1% KOH and 67 mM EDTA in 70% methanol containing chloro-ATP as an internal standard) was added to ∼0.5 g of each liver sample. The mixtures were promptly homogenized using an Omni-Tip TH with disposable, hard tissue homogenizer probes (Omni International). Aliquots of the homogenate were filtered by using 0.2-μm 96-well polypropylene filter plates (Varian Captiva). The filtrates were evaporated to dryness and reconstituted with an equal volume of 1 mM ammonium phosphate buffer (pH 7) prior to LC-MS/MS analysis. Plasma and liver samples were analyzed on a Sciex API-4000 (Applied Biosystems, Foster City, CA) LC-MS/MS instrument. Analytes in plasma samples were separated on a 4-μm, 150- by 2-mm Synergi Max-RP column (Phenomenex, Torrance, CA) using a mobile phase containing 0.2% formic acid and a linear gradient from 2% to 100% acetonitrile at a flow rate of 250 μl/min over 7 min. Analytes in liver samples were separated using a 50- by 2-mm by 2.5-μm Luna C_18_(2) HST column (Phenomenex, Torrance, CA). A multistage linear gradient from 10% (mobile phase A) to 50% acetonitrile (mobile phase B) in a mobile phase containing 3 mM ammonium formate (pH 5.0) with 10 mM dimethylhexylamine at a flow rate of 0.15 ml/min was used to elute the analytes.

### Antiviral EC_50_ determination.

The EC_50_s, defined as the concentration at which there was a measured 50% decrease in HCV replicon level, were determined in replicon assay as previously described (24). Briefly, replicon cells were seeded into 96-well plates. Compounds were serially diluted in DMSO at 200× final concentrations and then added to the assay plate. Luciferase expression was quantified after 3 days of incubation using a commercial luciferase assay (Promega, Madison, WI). Data were analyzed using GraphPad Prism 6.0 (La Jolla, CA). EC_50_s were calculated by nonlinear regression analysis using a sigmoidal dose-response variable slope equation (four-parameter logistic equation):

(1) $$Y=\frac{100}{1+10^{\left({\text{LogEC}}_{50}-\text{X}\right)\times \text{Hillslope}}}$$

*X* represents log of compound concentration and *Y* represents replicon replication as the percentage of DMSO control. EC_50_s were calculated as an average from at least three independent experiments.

### Western analysis for expression of CatA and CES1 in different cells.

Protein extract from the different cells were prepared by resuspending 2 × 10^6^ cells in 200 μl of 1× lysis buffer with 1× protease/phosphatase inhibitor cocktail (Cell Signaling, Danvers, MA) and incubating on ice for 20 min. Extracts were then centrifuged at 1,000 × *g* for 5 min to remove insoluble components. The supernatants were collected and normalized to 2 mg/ml protein after quantitation using the bicinchoninic acid protein assay kit (Thermo Scientific, Rockford, IL). For Western analysis, cell extract (50, 100, and 200 μg), human liver S9 (0.5 μg for CES1 and 10 μg for CatA), human intestinal S9 (10 μg), recombinant CatA (3.1 to 100 ng; 2-fold dilutions), and recombinant CES1 (1.6 to 50 ng; 2-fold dilutions) were resolved by SDS-PAGE (4 to 12% Bis-Tris NuPAGE; Life Technologies, Grand Island, NY) and transferred to nitrocellulose membranes using an iBlot gel transfer device (Life Technologies, Grand Island, NY). Nitrocellulose membranes were blocked with 10% nonfat dry milk in Tris-buffered saline–0.1% Tween 20 solution (TTBS) overnight at 4°C and incubated with either anti-CatA (0.2 μg/ml) or anti-CES1 (2 μg/ml) primary antibodies, followed by appropriately matched horseradish peroxidase-conjugated secondary antibodies (1:2,000 and 1:1,000, respectively) diluted in 5% milk–TTBS. Immunoreactive protein bands were visualized by enhanced chemiluminescence (Thermo Scientific, Rockford, IL) on a VersaDoc Imaging System and quantitated by densitometry under subsaturating exposure conditions using Quantity One v4.3.0 software (Bio-Rad, Hercules, CA). Cellular expression levels of CatA and CES1 were determined from a dilution series of recombinant CatA and CES1 that were fit by linear regression and resolved on the same polyvinylidene difluoride (PVDF) membrane(s).

### Pharmacokinetic analysis.

Noncompartmental pharmacokinetic parameters were calculated using WinNonlin 5.01 (Pharsight Corporation, Mountain View, CA). Equations for calculating hepatic extraction (*E_H_*; equation 2), fraction absorbed (*F_a_*; equation 3), and oral bioavailability (*F*; equation 4) have been presented previously (25).

(2) $$E_{H}=\frac{{\text{AUC}}_{\text{PO},\text{portal vein}}-{\text{AUC}}_{\text{PO},\text{jugular vein}}}{{\text{AUC}}_{\text{PO},\text{portal vein}}}$$

(3) $$F_{a}=\frac{F}{1-E_{H}}$$

(4) $$F=\frac{{\text{AUC}}_{\text{PO},\text{jugular vein}}÷{\text{Dose}}_{\text{PO}}}{{\text{AUC}}_{\text{IV}}÷{\text{Dose}}_{\text{IV}}}$$

### Statistical analysis.

Two-way analysis of variance (ANOVA) was conducted to evaluate if the difference between two compared groups is more than that expected by chance. The *P* values were calculated using GraphPad Prism (version 7.0).
